# Learn Less, Infer More: Learning in the Fourier Domain for Quantitative Susceptibility Mapping

**DOI:** 10.3389/fnins.2022.837721

**Published:** 2022-02-16

**Authors:** Junjie He, Lihui Wang, Ying Cao, Rongpin Wang, Yuemin Zhu

**Affiliations:** ^1^Key Laboratory of Intelligent Medical Image Analysis and Precise Diagnosis of Guizhou Province, College of Computer Science and Technology, Guizhou University, Guiyang, China; ^2^International Exemplary Cooperation Base of Precision Imaging for Diagnosis and Treatment, Department of Radiology, Guizhou Provincial People's Hospital, Guiyang, China; ^3^CREATIS, IRP Metislab, University of Lyon, INSA Lyon, CNRS UMR 5220, Inserm U1294, Lyon, France

**Keywords:** quantitative susceptibility mapping, deep learning, Fourier domain, magnetic susceptibility anisotropy, drug addiction

## Abstract

Quantitative susceptibility mapping (QSM) aims to evaluate the distribution of magnetic susceptibility from magnetic resonance phase measurements by solving the ill-conditioned dipole inversion problem. Removing the artifacts and preserving the anisotropy of tissue susceptibility simultaneously is still a challenge in QSM. To deal with this issue, a novel k-QSM network is proposed to resolve dipole inversion issues in QSM reconstruction. The k-QSM network converts the results obtained by truncated k-space division (TKD) into the Fourier domain as inputs. After passing through several convolutional and residual blocks, the ill-posed signals of TKD are corrected by making the network output close to the calculation of susceptibility through multiple orientation sampling (COSMOS)-labeled QSM. To evaluate the superiority of k-QSM, comparisons with several state-of-the-art methods are performed in terms of QSM artifacts removing, anisotropy preserving, generalization ability, and clinical applications. Compared to existing methods, the k-QSM achieves a 22.31% lower normalized root mean square error, 10.30% higher peak signal-to-noise ratio (PSNR), 33.10% lower high-frequency error norm, and 1.06% higher structural similarity. In addition, the orientation-dependent susceptibility variation obtained by k-QSM is significant, verifying that k-QSM has the ability to preserve susceptibility anisotropy. When the trained models are tested on the dataset from different centers, our k-QSM shows a strong generalization ability with the highest PSNR. Moreover, by comparing the susceptibility maps between healthy controls and drug addicts with different methods, we found the proposed k-QSM is more sensitive to the susceptibility abnormality in the patients. The proposed k-QSM method learns less—only to fix the ill-posed signals of TKD, but infers more—both COSMOS-like and anisotropy-preserving QSM results. Its generalization ability and great sensitivity to susceptibility changes can make it a potential method for distinguishing some diseases.

## 1. Introduction

Quantitative susceptibility mapping (QSM) is a magnetic resonance imaging (MRI) technique that measures magnetic susceptibility values in tissue from MRI phase measurements (Wang and Liu, 2014; Haacke et al., 2015). Phase values can reveal the sensitivity of tissues to a static magnetic field. The sensitivity is determined by magnetic susceptibility, whose contributors include biometals and molecules, for example, calcium, iron, myelin, and lipids (Feng et al., 2021). Tissue susceptibility is also a significant biomarker in pathological analysis. QSM has shown great potential for studying a number of neurodegenerative diseases, such as multiple sclerosis (MS) (Langkammer et al., 2013; Chen et al., 2014), Alzheimer's disease (AD) (Acosta-Cabronero et al., 2013; Ayton et al., 2017), intracranial hemorrhage (IH) (Ayton et al., 2017), and Parkinson's disease (PD) (van Bergen et al., 2015). However, QSM reconstruction is non-trivial and requires several processing steps involving phase unwrapping (Abdul-Rahman et al., 2007), background field removal (Schweser et al., 2011; Zhou et al., 2014), and dipole inversion, which is an ill-conditioned problem and a source of streaking artifacts (Salomir et al., 2003) due to the singularity in the dipole kernel and the limitation of phase measurements in multiple orientations (Deistung et al., 2016).

Different strategies have been proposed to process dipole inversion using additional measurements or numerical strategies. Truncated k-space division (TKD) (Shmueli et al., 2009) calculates susceptibility directly in k-space. It is uncomplicated, but finding an appropriate truncate value is challenging due to the susceptibility values and artifact reduction. The calculation of susceptibility through multiple orientation sampling (COSMOS) (Liu et al., 2008) is a high-fidelity approach to generate susceptibility maps and is regarded as the gold standard for QSM reconstruction. COSMOS, however, is not patient-friendly for clinical implementation because of the acquisition of at least three different orientations. It assumes isotropic magnetic susceptibility and contains little information regarding anisotropic tissue properties (Lee et al., 2010; Li W. et al., 2012; Wharton and Bowtell, 2012). Additionally, the dipole inversion problem can be solved in a spatial domain by virtue of priori information and optimum solutions, such as MEDI (Liu et al., 2011), iLSQR (Li et al., 2015), and STAR-QSM (Wei et al., 2015), which only obtain the numerical solution of the susceptibility value, require fine-tuned parameters, and suffer from artifacts or oversmoothing.

In recent years, deep-learning-based QSM algorithms have shown the ability to approximate dipole inversion and generate high-quality QSM reconstructions with less time consumption (Jin et al., 2017). Yoon et al. (2018) proposed QSMnet to train a 3D U-Net Ronneberger et al. (2015) to infer high-quality COSMOS QSM from single orientation scanning tissue phase data. Its successor QSMnet^+^ improved the generalization performance by data augmentation to surmount the underestimation of high susceptibility values in the brain (Jung et al., 2020). Instead of training *in vivo* data, it can be trained on synthetic data, as indicated by DeepQSM (Bollmann et al., 2019). Chen et al. (2020) advocated QSMGAN, a generative adversarial network, utilizing the power of adversarial learning for QSM reconstruction. Lai et al. (2020) presented a learned proximal convolutional neural network (LP-CNN) to perform dipole inversion in an iterative proximal gradient descent fashion. Gao et al. (2020) proposed an improved U-Net framework, namely xQSM, for dipole inversion by incorporating octave convolution (OctConv) (Chen et al., 2019) layers. Feng et al. (2021) proposed an STI-based deep learning architecture for single-orientation QSM, referred to as MoDL-QSM, which can preserve the nature of anisotropic magnetic susceptibility in brain white matter (WM). All of these deep-learning-based methods are either anisotropy free because of being COSMOS-labeled or lack of accurate susceptibility values for being numerical QSM-labeled, for example, STAR-QSM. In addition, all these models trained for dipole inversion are in spatial space instead of k-space directly, which means they have to learn the entire field-magnetic relationship and, thus, may result in more error accumulation.

In this study, a deep convolutional neural network (DCNN) (Jin et al., 2017) network, called k-QSM, is proposed to resolve the QSM dipole inversion problem learned in the Fourier domain (or k-space), with the aim to reconstruct COSMOS-like QSM while maintaining magnetic susceptibility anisotropy in WM. The k-QSM intended to fix the ill-posed signals of TKD and retained numerous correct signals simultaneously, which allows us to learn less—only to fix the ill-posed signals of TKD, but to infer more—both COSMOS-like and anisotropy-preserving QSM results.

## 2. Materials and Methods

### 2.1. Theory of QSM Dipole Inversion

Under the effect of an external main magnetic field, the varying susceptibility distribution(χ) influences the local magnetic field due to the whirling electrons in MRI. As shown from Maxwell magnetostatic equations and the Lorentz correction for media effects, the susceptibility distribution alters the local field along the uniform magnetic field (Marques and Bowtell, 2005), conforming to the following equation:

(1) $$\begin{array}{l} \delta_{B}\left(\vec{r}\right)=\frac{1}{4\pi }∭\chi \left(\vec{r}{\;}^{\prime }\right)\frac{3\cos^{2}\alpha -1}{|\vec{r}{\;}^{\prime }-\vec{r}{|}^{3}}d^{3}\vec{r}{\;}^{\prime }=\frac{3\cos^{2}\alpha -1}{4\pi |\vec{r}{|}^{3}}⊗\chi \left(\vec{r}\right) \end{array}$$

where $\vec{r}$ is the spatial coordinate, α is the angle between the applied field and $\vec{r}{\;}^{\prime }-\vec{r}$, $\delta_{B}\left(\vec{r}\right)=\left[B\left(\vec{r}\right)-B_{0}\right]/B_{0}$ represents the relative difference of the magnetic field, *B* is the local magnetic field component along the main magnetic field (Li and Leigh, 2004), and ⊗ denotes the 3D convolution operator.

In the Fourier domain, this convolution relationship can be simplified as a point-wise multiplication with a dipole kernel (Salomir et al., 2003):

(2) $$\begin{array}{l} \Delta_{B}\left(\vec{k}\right)=\left(\frac{1}{3}-\frac{k_{p}^{2}}{k^{2}}\right)\cdot X\left(\vec{k}\right)=D\left(\vec{k}\right)\cdot X\left(\vec{k}\right) \end{array}$$

where Δ_*B*_ and *X* are the Fourier transforms of δ_*B*_ and χ, respectively, $\vec{k}$ is the k-space coordinate of $\vec{r}$, *k* is the magnitude of $\vec{k}$, $D\left(\vec{k}\right)=1/3-k_{p}^{2}/k^{2}$ is the dipole kernel, and $k_{p}=\vec{k}\cdot \vec{B_{0}}$ is the projection of $\vec{k}$ onto the direction of the main magnetic field. $\vec{B_{0}}$ is the main magnetic field orientation (B_0_ vector).

To calculate *X*, a direct point-wise division has been proposed (Marques and Bowtell, 2005) but is challenging in practice, requiring calculation of ${\left(1/3-k_{p}^{2}/k^{2}\right)}^{-1}$, which is ill-conditioned around the zeros on two conical surfaces at approximately 54.7° from the main magnetic field (Liu et al., 2008). Numerous traditional methods have been proposed to solve this ill-conditioned problem, among which only TKD and COSMOS are analytical solutions and have been evaluated in k-space. TKD measures QSM by:

(3) $$\begin{array}{l} X_{TKD_{thr}}\left(\vec{k}\right)=\frac{\text{sign}\left(D\left(\vec{k}\right)\right)}{\max\left(|D\left(\vec{k}\right)|,thr\right)}\cdot \Delta_{B}\left(\vec{k}\right) \end{array}$$

where *thr* is the truncated value chosen to achieve a reasonable artifact level (Shmueli et al., 2009). COSMOS determines the susceptibility value using multiple measurements from multiple sampling orientations at a given location in the Fourier domain $X\left(\vec{k}\right)$ (Liu et al., 2008):

(4) $$\begin{array}{l} \left[\begin{array}{c} D_{1}\left(\vec{k}\right)\\ D_{2}\left(\vec{k}\right)\\ ...\\ D_{n}\left(\vec{k}\right) \end{array}\right]\cdot X\left(\vec{k}\right)=\left[\begin{array}{c} \Delta_{B_{1}}\left(\vec{k}\right)\\ \Delta_{B_{2}}\left(\vec{k}\right)\\ ...\\ \Delta_{B_{n}}\left(\vec{k}\right) \end{array}\right]. \end{array}$$

When *n* ≥ 3, the above criterion can be fulfilled for every point in the Fourier domain, which has been proven (Liu et al., 2008). In this study, the following equation is used to calculate the COSMOS QSM:

(5) $$\begin{array}{l} X_{COSMOS}\left(\vec{k}\right)=\frac{\sum_{i=1}^{n}\Delta_{B_{i}}\cdot D_{i}\left(\vec{k}\right)}{\sum_{i=1}^{n}D_{i}^{2}\left(\vec{k}\right)}. \end{array}$$

**Figure 1:**
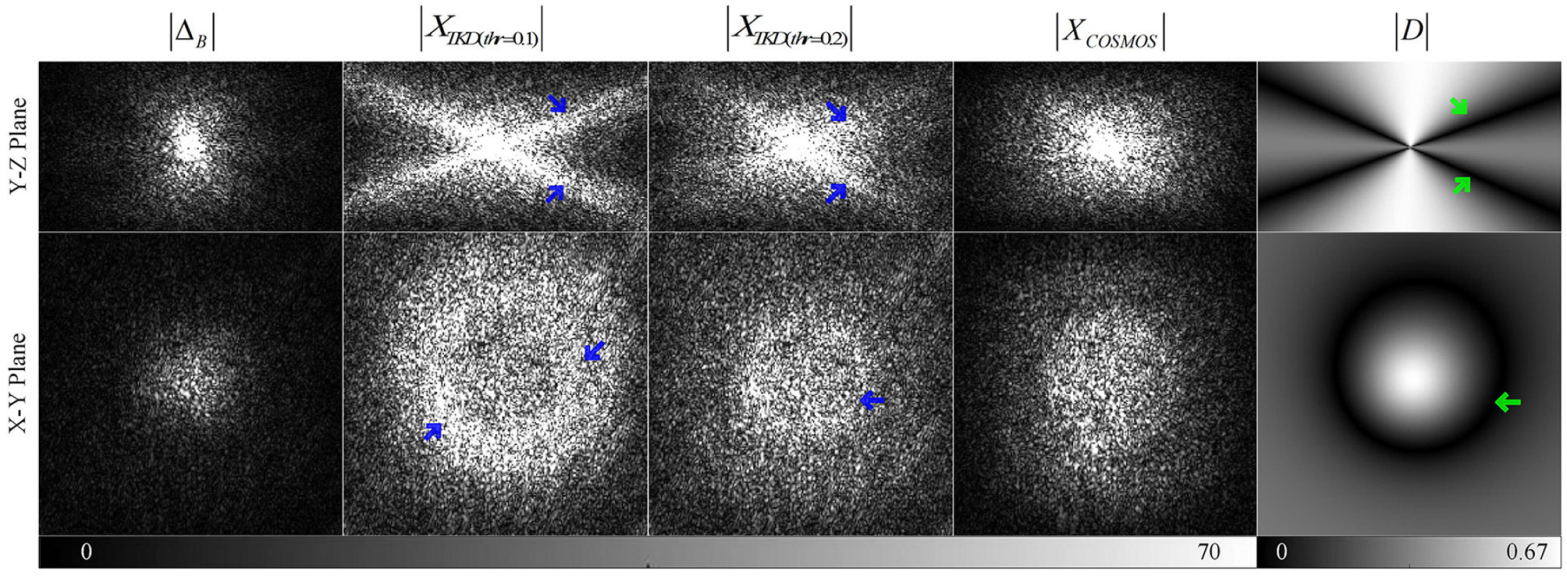
Comparison of the k-space data of Field Map, TKD QSM, calculation of susceptibility through multiple orientation sampling (COSMOS) QSM, and dipole kernel in Y-Z and X-Y plane views (the X-Z plane is almost the same as the Y-Z plane) with green arrows indicating zeros in dipole kernel and blue arrows marking the ill-posed signals due to the truncation.

Figure 1 illustrates the difference between TKD and COSMOS in k-space, which shows extremely high signals of *X*_*TKD*_ (blue arrows) around $D\left(\vec{k}\right)\approx 0$ (green arrows). This causes the streaking artifacts in the TKD QSM. The original paper suggested *thr* = 0.2 to reduce noise amplification and streaking artifacts (Shmueli et al., 2009) but resulted in underestimating susceptibility values (red arrows in **Figure 3**). Inspired by the difference between TKD and COSMOS, the existence of problematic values around $D\left(\vec{k}\right)\approx 0$, a deep-learning method is proposed to restore the ill-posed values.

### 2.2. k-QSM Deep Learning Architecture

**Figure 2:**
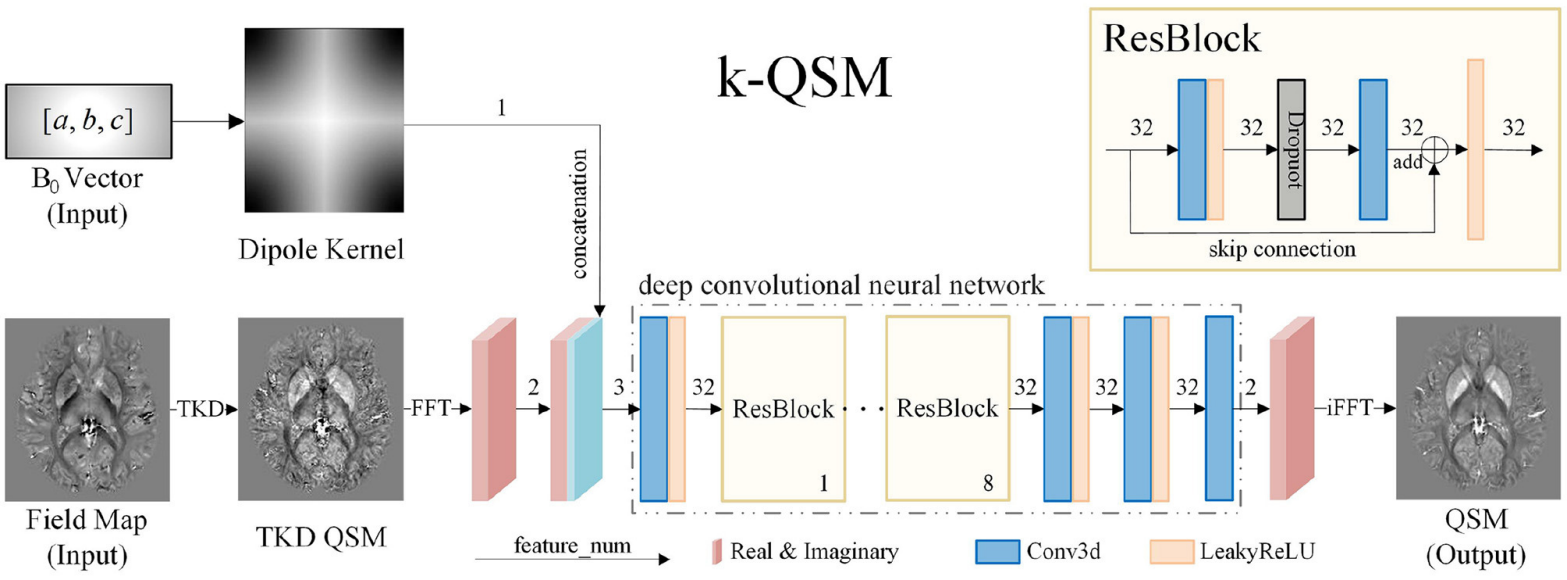
The proposed k-QSM network architecture. The input of the model is B_0_ vector and the field map, and the output is quantitative susceptibility mapping (QSM). The input and output of the deep convolutional neural network (DCNN) are both k-space data.

The architecture of the k-QSM is illustrated in Figure 2. The model takes the B_0_ vector and magnetic field maps as input. The B_0_ vector is used to generate the dipole kernel as a forward operator using Equation (2). The field map is first converted into QSM using the TKD method. A fast Fourier transform (FFT) is then applied to the TKD QSM, resulting in real and imaginary parts as two features, concatenated with a dipole kernel as the input of the DCNN. The output of the DCNN consists of two features—the real and imaginary parts—which are used to calculate the QSM through inverse fast Fourier transform (iFFT). The entire process can be expressed as follows:

(6) $$\begin{array}{l} \{\begin{array}{c} c=iFFT\left(DCNN_{\theta }\left(D,X_{TKD}\right)\right)\\ QSM=\text{abs}\left(c\right)\times \text{sign}\left(\text{real}\left(c\right)\right) \end{array} \end{array}$$

where θ represents the learned parameters of the DCNN, in which a ConvBlock (3D convolutional layer + LeakyReLU layer) is first used to extract local features among adjacent signals in the k-space map of TKD QSM, and eight ResBlocks (Kim et al., 2016), composed of ConvBlock, Dropout layer, Convolutional layer, and residual connection, are then used to extract global features and avoid the vanishing gradient issue (He et al., 2016). Finally, two consecutive ConvBlocks and a convolutional layer are implemented for fusing the feature maps to form the real and imaginary parts of k-space map of reconstructed QSM. Note that, in all the convolutional layers, the *kernel size* = 3, *stride* = 1, and *padding* = 1. In the LeakyReLU layers, we set *negative slope* = 0.1. In the ResBlocks, the introduction of Dropout layer can prevent overfitting (Srivastava et al., 2014) and enhance the generalization ability (Dahl et al., 2013) of our network; therefore, we set *drop rate* = 0.2.

To train the k-QSM model, two loss functions were designed to optimize the network over the training dataset. The first was the mean squared error (MSE) to fix the ill-signals in TKD compared to COSMOS as defined by:

(7) $$\begin{array}{l} loss_{MSE}=\frac{1}{N}\overset{N}{\underset{i=1}{\sum }}{\left\|X_{label_{i}}-\hat{X_{i}}\right\|}_{2}^{2} \end{array}$$

where *N* is the size of the training set, *X*_*label*_*i*__ is COSMOS in k-space, $||\cdot |{|}_{2}^{2}$ is the squared L2 norm, and $\hat{X_{i}}$ is the output of the DCNN. The second was the mean absolute error (MAE) or L1 loss served as a consistency loss to maintain the consistency between the output $\hat{X_{i}}$ and the input *X*_*TKD*_*i*__:

(8) $$\begin{array}{l} loss_{L1}=\frac{1}{N}\overset{N}{\underset{i=1}{\sum }}|X_{TKD_{i}}{|}_{cond}-\hat{X_{i}}{|}_{cond}| \end{array}$$

where *cond* = *abs*(*D*) ≥ *thr* indicates the non-ill-posed regions in a dipole kernel. The total loss was designed as the weighted sum of the two losses:

(9) $$\begin{array}{l} loss_{T}=\omega_{1}\cdot loss_{MSE}+\omega_{2}\cdot loss_{L1} \end{array}$$

where the weights were determined as ω_1_ = 1 and ω_2_ = 1 empirically.

The k-QSM model parameters were optimized using the Adam optimizer (Kingma and Ba, 2014) with an initial learning rate of 10^−4^, which dropped to 90% every 25 epochs. The training data were cropped patches of size 64^3^ in the Fourier domain with an overlap 50% between adjacent patches owing to the limitation of GPU memory. The proposed network was implemented using Python 3.7 and Pytorch 1.8.1, and trained on NVIDIA Tesla A100 GPU. The source codes have been published at https://github.com/TyrionJ/k-QSM.

### 2.3. Data Acquisition and Processing

The proposed k-QSM model was trained and tested on data from the F. M. Kirby Research Center for Functional Brain Imaging, which provides 36 MR phase measurements at 7T Scanner (Philips Achieva) with a voxel size of 1 × 1 × 1*mm*^3^ on eight healthy subjects, including five orientations for four subjects with *FOV* = 224 × 224 × 100*mm*^3^, *TR* = 28*ms*, *TE*_1_/Δ*TE* = 5/5*ms*, and 5 *echoes*; four orientations for three subjects with *FOV* = 224 × 224 × 100*mm*^3^, *TR* = 45*ms*, *TE*_1_/Δ*TE* = 2/5*ms*, and 9 *echoes*; and four orientations for one subject with *FOV* = 224 × 224 × 100*mm*^3^, *TR* = 45*ms*, *TE*_1_/Δ*TE* = 2/5*ms*, and 16 *echoes*. Six subjects were used for training, and the training dataset was also augmented by exerting different dipole kernels on convolution with COSMOS QSM to obtain local field maps. The remaining one subject was used for validation to prevent overfitting, and the other one subject was used for performance testing and measurement of magnetic susceptibility anisotropy.

To verify the robustness and generalization ability of the trained k-QSM model, multiple healthy and pathological datasets from different centers were used. Data on healthy subjects were *in vivo* brain data provided for the 2016 QSM Reconstruction Challenge (Langkammer et al., 2018), including 12 orientation measurements at 3T scanner (Siemens) on one healthy subject with *voxel size* = 1.06 × 1.06 × 1.06*mm*^3^, *FOV* = 170 × 170 × 170*mm*^3^, *TR* = 25*ms* and *TE* = 35*ms*. Patient data were cited from the study of Feng et al. (2021), including data of patients with multiple sclerosis and intracranial hemorrhage.

To further evaluate our proposed method in practical applications, we tested it on in-house dataset acquired from 15 patients with heroin addiction and 15 age- and sex-matched healthy volunteers using a 3T GE scanner with *voxel size* = 1.06 × 1.06 × 1.06*mm*^3^, *FOV* = 256 × 256 × 136*mm*^3^, *TR* = 28*ms*, *TE*_1_/Δ*TE* = 3.276/2.352*ms* and 16 *echoes*. Signed informed consent was obtained from all the participants. This dataset was used to verify the variation in susceptibility among different gray matter regions between heroin addicts and healthy individuals. Multiple steps were required to preprocess phase images, including Laplacian-based phase unwrapping (Schofield and Zhu, 2003), brain extraction with FSL BET (Smith, 2002), V-SHARP for background field removal (Özbay et al., 2017), echo averaging, and conventional QSM reconstruction using the STAR-QSM (Wei et al., 2015) pipeline. For region of interest (ROI) analysis, registration was applied to all subjects according to the automated anatomical labeling (AAL) template (Tzourio-Mazoyer et al., 2002). Registration was first conducted on magnitude images, generating transforms to move from moving (QSM) to fixed (Atlas) images.

## 3. Results

### 3.1. Performance of the k-QSM

**Figure 3:**
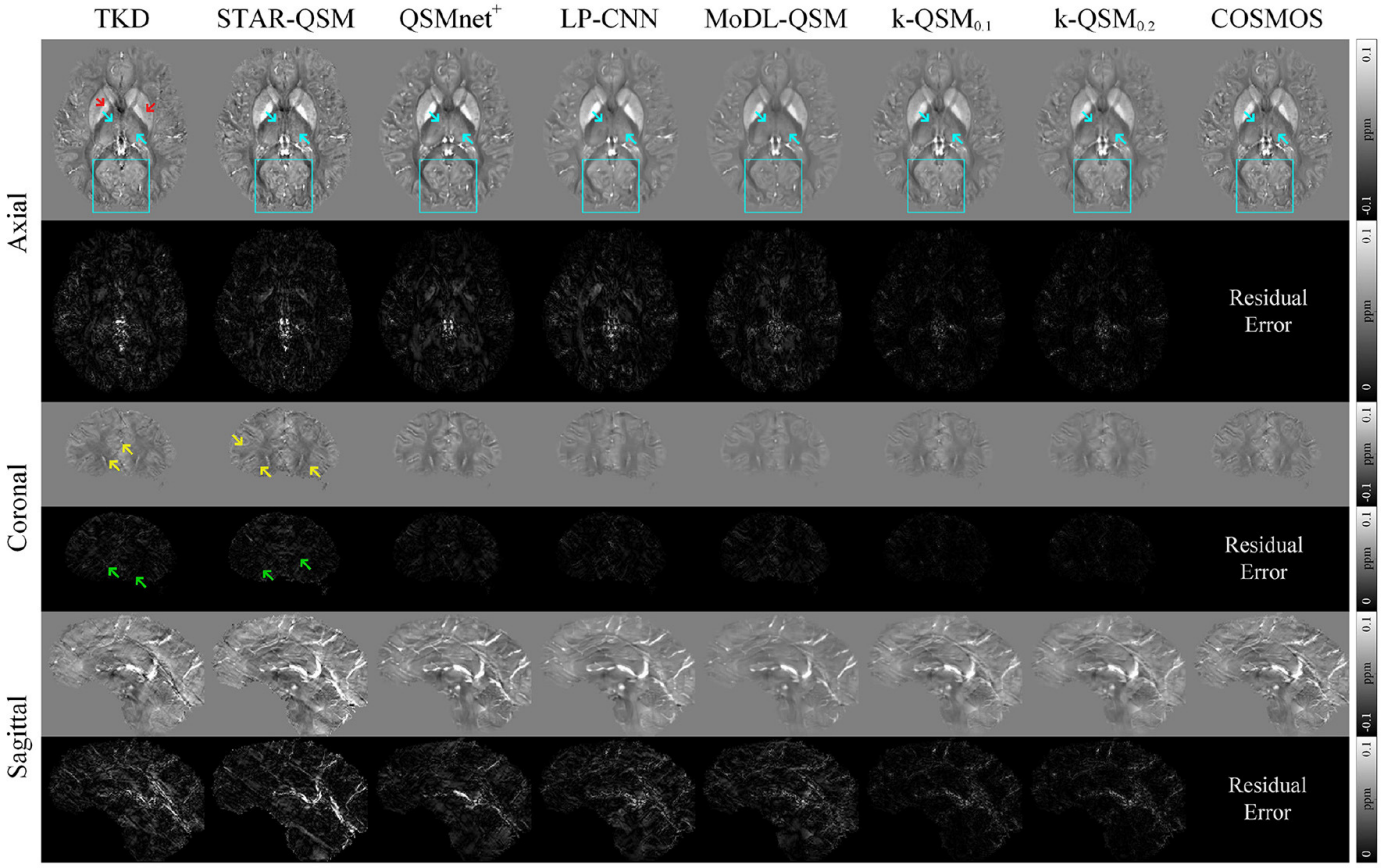
Three orthogonal views of quantitative susceptibility mapping (QSM) reconstruction (average of five orientation samplings) using different methods on data from the FM Kirby Research Center. The k-QSM result shows more details and textures (cyan arrows and boxes) than other deep learning methods. Truncated k-space division (TKD) (thr = 0.2) and STAR-QSM can reconstruct the details but with insufferable streak artifacts (yellow arrows). Red arrows mark lower susceptibility of TKD than COSMOS. Green arrows indicate artifacts in residual error maps.

Figure 3 displays the results and a comparison of the three orthogonal planes with residual error maps. QSMnet^+^, LP-CNN, and MoDL-QSM presented here were all retrained using labels calculated from our training data. We denote k-QSM_*T*_ as training and testing with truncated value of T. The figure shows that k-QSM_0.1_ and k-QSM_0.2_ share similar results even though took different truncated values. Cyan arrows indicate that the results of k-QSM show more high-frequency details of the thalamus than those of QSMnet^+^, LP-CNN, and MoDL-QSM, which are too smooth to reveal textures. In the occipital lobe regions, outlined by cyan boxes, the results of k-QSM and QSMnet^+^ illustrate more COSMOS-like folds in detail than LP-CNN and MoDL-QSM. Although TKD and STAR-QSM can also measure the details to some degree, the results are accompanied by numerous unacceptable streak artifacts, marked by yellow arrows. Briefly, k-QSM shows the least residual errors regarding COSMOS among the QSM reconstruction methods. It achieves a balance of artifact reduction and detail measurement, and more COSMOS-like details and susceptibility contrasts than other methods.

Table 1 gives the results of the proposed k-QSM and other methods in terms of quantitative metrics of peak signal-to-noise ratio (PSNR), normalized root mean square error (NRMSE), high-frequency error norm (HFEN), and structural similarity (SSIM). For all criteria, k-QSM_0.1_ shows better performance than the other methods with the lowest NRMSE (38.65%), the highest PSNR (42.08 dB), the second lowest HFEN (29.53%), and the highest SSIM (99.40%). Compared to the best previous method—MoDL-QSM—the k-QSM achieves a 22.31% lower NRMSE, 10.30% higher PSNR, 33.10% lower HFEN, and 1.06% higher SSIM. Besides, the performances of k-QSM_0.1_ and k-QSM_0.2_ stay close.

**Table 1:** Comparison of quantitative performance metrics on results from different quantitative susceptibility mapping (QSM) reconstruction methods referenced to calculation of susceptibility through multiple orientation sampling (COSMOS) on data from the FM Kirby Research Center.

**Methods**	**NRMSE (%)**	**PSNR (dB)**	**HFEN (%)**	**SSIM (%)**
TKD	70.26	34.11	111.70	96.00
STAR-QSM	48.62	36.95	63.07	97.64
QSMnet^+^	48.37	39.41	49.97	96.94
LP-CNN	54.17	38.22	50.25	98.32
MoDL-QSM	49.75	38.15	44.14	98.36
**k-QSM**_**0.1**_	**38.65**	**42.08**	29.53	**99.40**
k-QSM_0.2_	39.35	41.92	**29.29**	99.38

*Bold values indicate the best performance metrics*.

**Figure 4:**
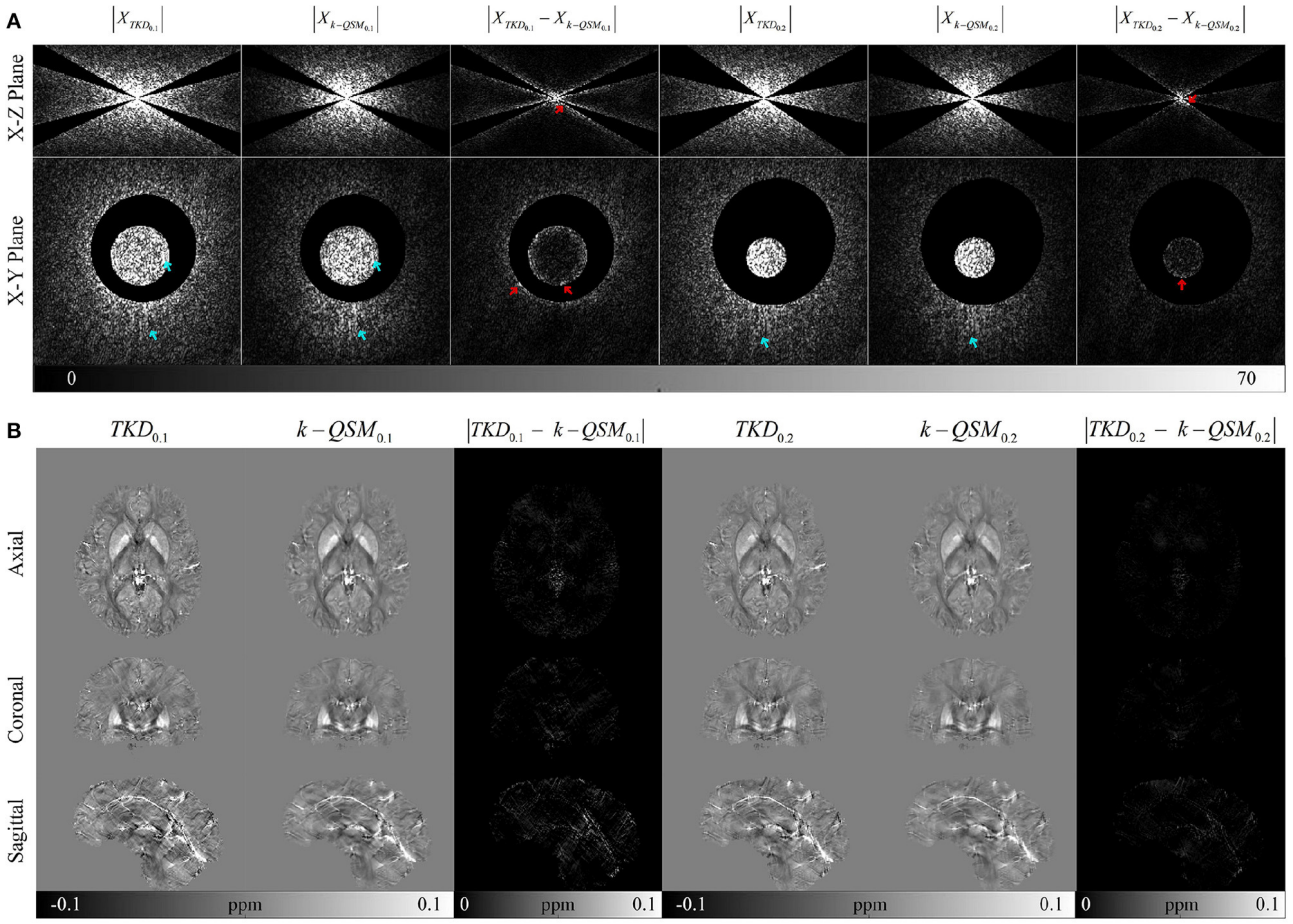
Comparison on signals of k-QSM output and truncated k-space division (TKD) in non-ill-posed regions with error residual views. **(A)** The signals in the Fourier domain, with cyan arrows indicating the similarity between TKD and k-QSM, and red arrows marking the differences around the boundaries. **(B)** The spatial domain data of **(A)**.

To verify the effect of consistency loss, we conducted experiment on TKD and k-QSM in the non-ill-posed regions of dipole kernel and the results are shown in Figure 4. There are a majority of signals (cyan arrows) of k-QSM results closer to those of TKD in the non-ill-posed areas as shown in Figure 4A, whereas some differences exist on the boundaries (red arrows). Figure 4B displays TKD and k-QSM results in the spatial domain from non-ill-posed signals. The results of k-QSM show less streak artifacts than TKD.

### 3.2. Magnetic Susceptibility Anisotropy in White Matter

QSM originates from MRI phase measurements, which have been found to depend on the orientation of fibers with respect to the main magnetic field (Denk et al., 2011). These effects can be ascribed to the orientation-dependent magnetic susceptibility anisotropy (MSA) (Wisnieff et al., 2013). Thus, it is appropriate for a QSM reconstruction method to preserve the MSA of brain tissues in the WM. Following Li X. et al. (2012), deep WM regions including the posterior limb of the internal capsule (PLIC) and posterior thalamic radiation (PTR) were selected to verify that the proposed k-QSM has the ability to preserve the MSA in WM.

**Figure 5:**
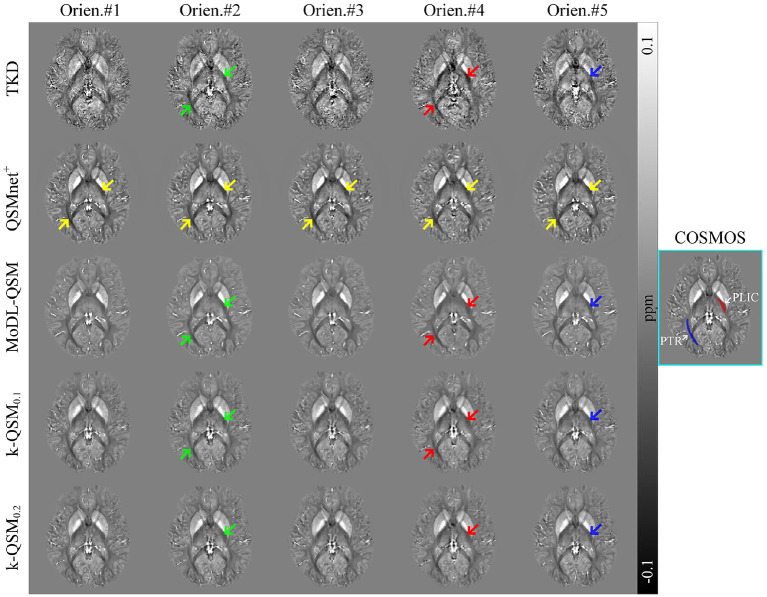
Axial views of quantitative susceptibility mapping (QSM) reconstructed from the same subject in five head orientations. Posterior limb of the internal capsule (PLIC) (red) and posterior thalamic radiation (PTR) (blue) are marked in calculation of susceptibility through multiple orientation sampling (COSMOS). Green, red, and blue arrows indicate the susceptibility anisotropy in white matter, and yellow arrows reveal the anisotropy free regions. Truncated k-space division (TKD) truncated value is 0.2.

Figure 5 illustrates the comparison of the reconstructed QSM in five head orientations using the TKD, QSMnet^+^, MoDL-QSM, and k-QSM methods. QSMnet^+^ and MoDL-QSM were tested using the pre-trained model provided by the authors to evaluate the ability to preserve the MSA of PLIC and PTR regions (marked in red and blue, respectively, in the COSMOS result outlined by the cyan box). The results of TKD, MoDL-QSM, and k-QSM_0.1_ show susceptibility variation in five orientations in PLIC and PTR, and the results of k-QSM_0.2_ show those only in PLIC. Red arrows indicate lower susceptibility values for PLIC and PTR than those marked by green arrows. Blue arrows indicate lower susceptibility values for PLIC than green arrows. Yellow arrows reveal the visually indistinguishable consistent susceptibility of PLIC and PTR in five orientations reconstructed by QSMnet^+^.

Table 2 shows the *p*-values obtained with various statistical tests, Levene (Brown and Forsythe, 1974), ANOVA (Sthle and Wold, 1989), and Kruskal–Wallis (KW) test (McKight and Najab, 2010), which are used to verify significantly different susceptibility variations of QSM reconstruction in five orientations by each method in regions of PLIC and PTR, as marked in Figure 5. The Levene test verifies the null hypothesis that all input samples are from populations with equal variances, which is required by ANOVA. The ANOVA test is valid if the result of Levene test is not significantly different (*p* > 0.05); otherwise, the KW test is valid, and the valid *p*-values are shown in bold. It can be summarized that the results of QSMnet^+^ were significantly the same in the five orientations tested by ANOVA in PLIC (*p* = 0.23) and KW in PTR (*p* = 0.21), and those of TKD, MoDL-QSM, and k-QSM_0.1_ were significantly different (*p* < 0.05) in PLIC and PTR. The results of k-QSM_0.2_ were only significantly different (*p* = 0.0049) in PLIC.

**Table 2:** Comparison of p-values of Leven, ANOVA, and Kruskal–Wallis (KW) tests on the results of five orientations using several methods in regions of posterior limb of the internal capsule (PLIC) and posterior thalamic radiation (PTR) of white matter.

	**PLIC**			**PTR**		
**Methods**	**Levene**	**ANOVA**	**KW**	**Levene**	**ANOVA**	**KW**
TKD	0.016	6.7 × 10^-32^	**6.8 × 10** ^**−21**^	6.5 × 10^-5^	4.5 × 10^-6^	**2.5 × 10** ^**−6**^
QSMnet^+^	0.011	0.211	**0.233**	0.81	**0.22**	0.21
MoDL-QSM	0.0015	6.3 × 10^-4^	**2.8 × 10** ^**−5**^	0.61	**0.0053**	0.0064
k-QSM_0.1_	0.0075	2.6 × 10^-6^	**5.4 × 10** ^**−6**^	0.51	**0.012**	0.034
k-QSM_0.2_	0.028	0.0018	**0.0049**	0.81	**0.14**	0.19

*Bold numbers indicate valid p-values*.

All these results show that the proposed k-QSM_0.1_ can preserve the MSA as well as TKD and MoDL-QSM, as the results of these methods display orientation-dependent susceptibility variation in the PLIC and PTR regions of WM, whereas QSMnet^+^ loses the MSA during QSM reconstruction and k-QSM_0.2_ loses parts of the MSA.

### 3.3. Generalization of the k-QSM

The generalization ability of a deep learning model on different datasets is crucial to its practical application. The trained k-QSM model was tested for the generalization ability on the data of healthy subjects (2016 QSM Reconstruction Challenge) and patients with MS and hemorrhage (cited from the study of Feng et al., 2021), which were not used for training.

**Figure 6:**
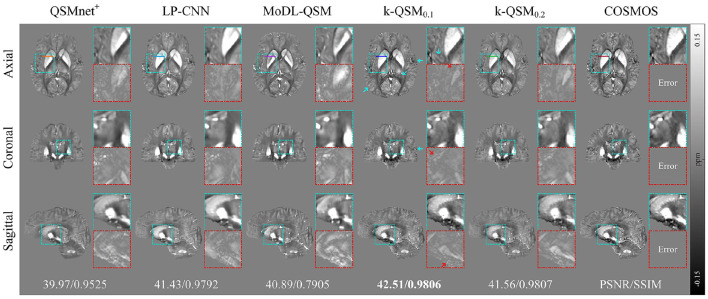
Three orthogonal views of reconstructed quantitative susceptibility mapping (QSM) (average of 12 orientation samplings) using different deep-learning methods, with zoomed-in details (cyan boxes) and errors (red boxes), and global peak signal-to-noise ratio (PSNR) and structural similarity (SSIM) with respect to calculation of susceptibility through multiple orientation sampling (COSMOS) reported below. Red arrows mark the lower error of k-QSM and cyan arrows indicate richer details.

Figure 6 displays the results of different deep-learning-based QSM reconstruction methods tested on the data of healthy individuals from three orthogonal views. The k-QSM_0.1_ achieves better results than k-QSM_0.2_. The proposed k-QSM outperforms the others in visualizing zoomed-in details (cyan boxes) and error maps (red boxes) related to COSMOS, with red arrows indicating lower errors of the k-QSM_0.1_ result and cyan arrows marking stronger contrast and richer details, which are more COSMOS like. Besides, the k-QSM_0.1_ achieves almost the best performance metrics with PSNR = 42.51 dB and SSIM = 98.06%, which are 14.0% better than those of MoDL-QSM, 1.4% better than those of LP-CNN, and 4.7% better than those of QSMnet^+^ on average.

**Figure 7:**
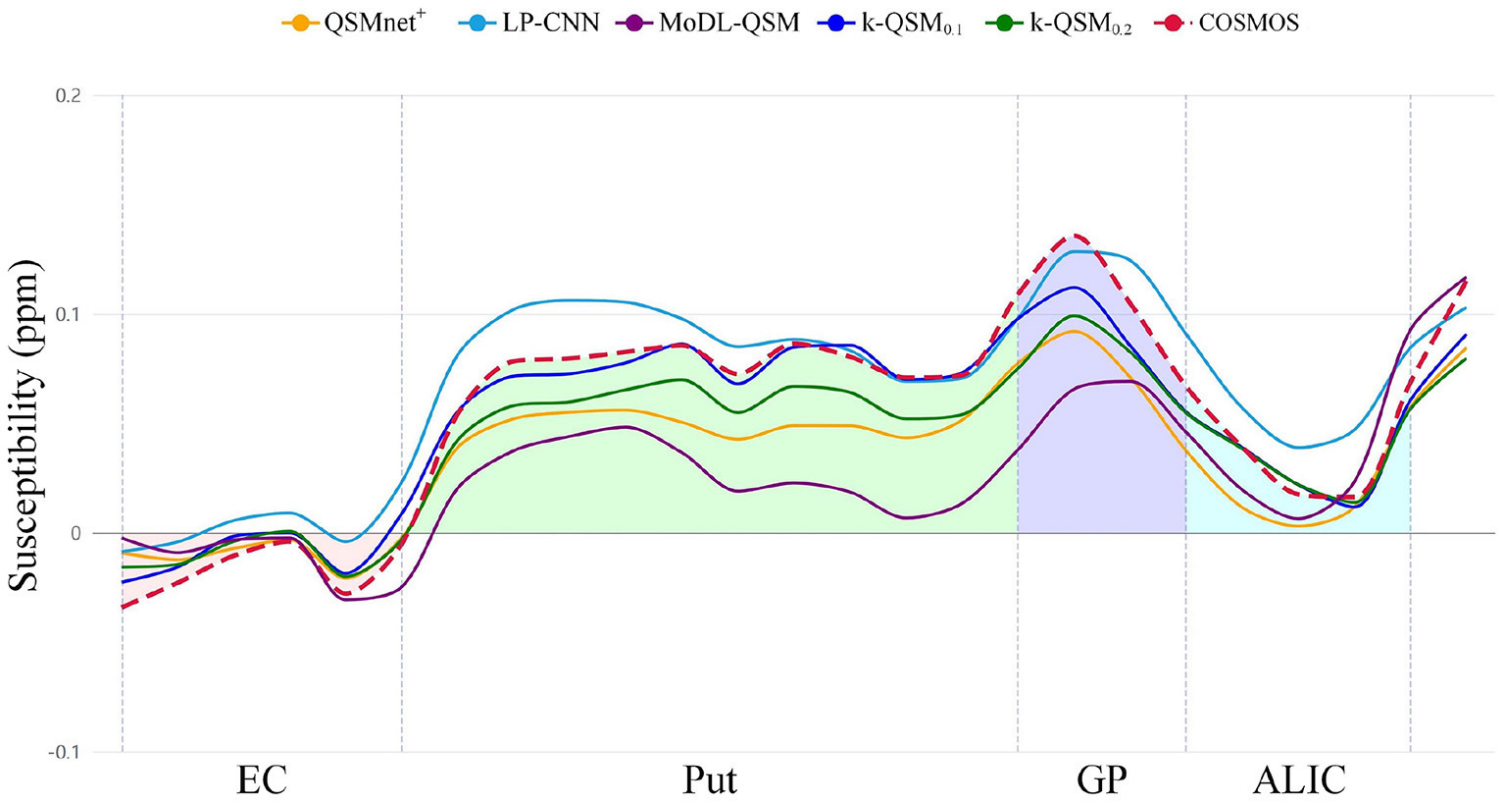
The profiles of susceptibility values calculated along the lines outlined in Figure 5 crossing external capsule (EC), putamen (Put), globus pallidus (GP), and anterior limb of internal capsule (ALIC) are plotted in the chart with color regions enclosed by calculation of susceptibility through multiple orientation sampling (COSMOS) and the axis.

Figure 7 shows that k-QSM_0.1_ and k-QSM_0.2_ produce a similar variation tendency of susceptibility values along the line profiles crossing the external capsule (EC), putamen (Put), globus pallidus (GP), and anterior limb of internal capsule (ALIC) compared to COSMOS, but k-QSM_0.2_ underestimates susceptibility in putamen and globus pallidus (24.6% lower than COSMOS on average). Meanwhile, QSMnet^+^ underrates susceptibility values (34.0% lower) in the four regions and MoDL-QSM undervalues that in Put (67.6% lower) and GP (47.5% lower). In contrast, the LP-CNN overestimates the susceptibility with values 26.4% higher than that of COSMOS.

Statistical metrics are shown in Table 3 of the MAE (Willmott and Matsuura, 2005), RMSE, and Pearson's correlation coefficient (PCC) between a deep-learning method and COSMOS. Our k-QSM_0.1_ achieves the best performance with MAE and RMSE values 75.68% better than MoDL-QSM, 50.74% better than LP-CNN, and 62.27% better than QSMnet^+^ on average, and with PCC values 28.57% better than MoDL-QSM, 2.06% better than LP-CNN, and 1.02% better than QSMnet^+^.

**Table 3:** Mean absolute error (MAE), root mean square error (RMSE), and Pearson's correlation coefficient (PCC) of line profiles outlined in Figure 6 with respect to calculation of susceptibility through multiple orientation sampling (COSMOS).

**Methods**	**QSMnet^**+**^**	**LP-CNN**	**MoDL-QSM**	**k-QSM_**0.1**_**	**k-QSM_**0.2**_**
MAE	0.022	0.017	0.033	**0.0078**	0.015
RMSE	0.025	0.019	0.040	**0.010**	0.018
PCC	0.98	0.97	0.77	**0.99**	0.99

*Bold values indicate the best performance metrics*.

**Figure 8:**
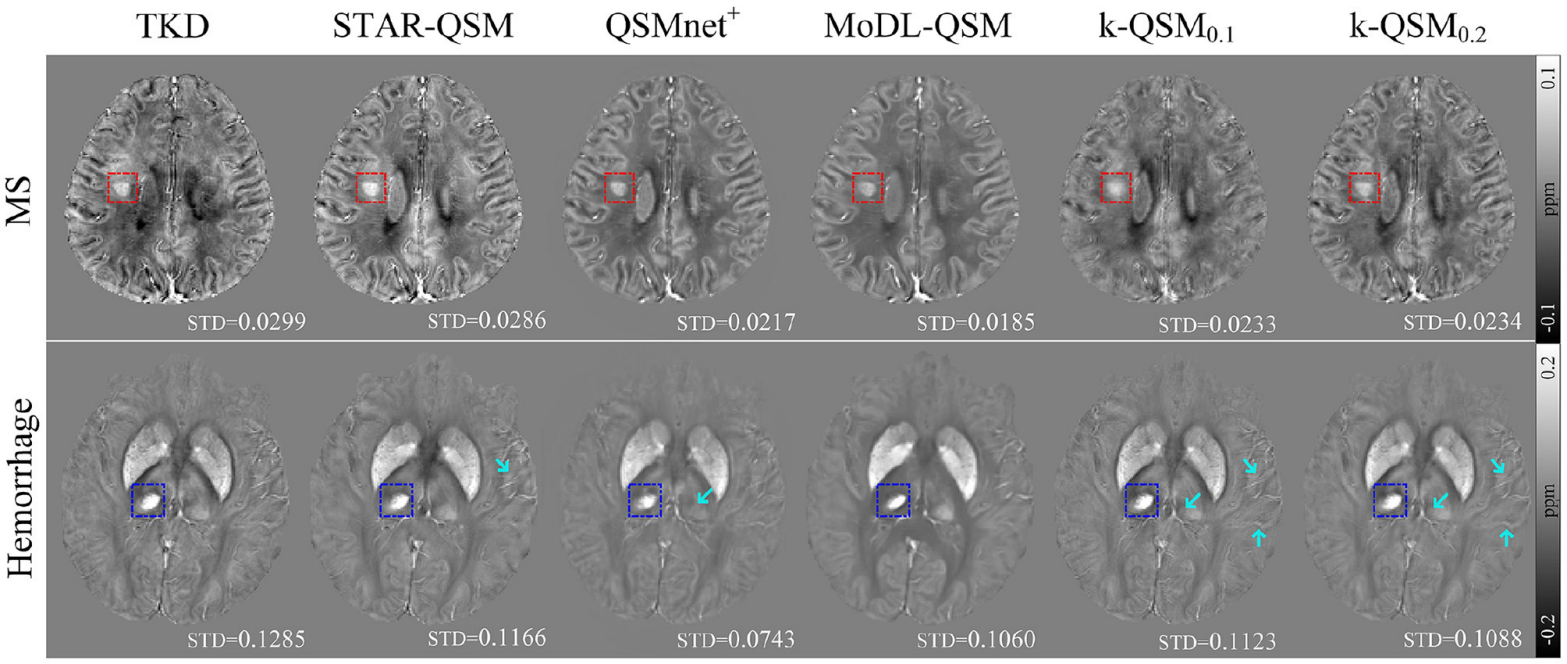
Axial views of quantitative susceptibility mapping (QSM) reconstructed using truncated k-space division (TKD) (thr = 0.2), STAR-QSM, QSMnet^+^, MoDL-QSM, and k-QSM on patients with MS (red boxes) and hemorrhage (blue boxes), and standard deviation (STD) values of the corresponding boxes are reported below. Cyan arrows mark richer details in the results of k-QSM and STAR-QSM.

Figure 8 shows the results of TKD, STAR-QSM, QSMnet^+^, MoDL-QSM, and k-QSM on the data of patients with MS and hemorrhage. All these QSM reconstruction methods showed the ability to detect the lesion regions of MS (red boxes) and hemorrhage (blue boxes). In addition, the k-QSM (both 0.1 and 0.2) and STAR-QSM can reconstruct a larger range of susceptibility value variations and richer details (marked by cyan arrows) than QSMnet^+^ and MoDL-QSM, whose results are too smooth to capture high-frequency information, which can be verified by the relatively small standard deviation values. Suffering from artifacts, the results of TKD showed a much higher standard deviation. By holding a moderate level of standard deviation (17.94 ± 4.72% lower than TKD and 15.25 ± 12.73% higher than MoDL-QSM), the proposed k-QSM achieves a balance between measuring high-frequency details and suppressing artifacts effectively.

### 3.4. Practical Application of the k-QSM

**Figure 9:**
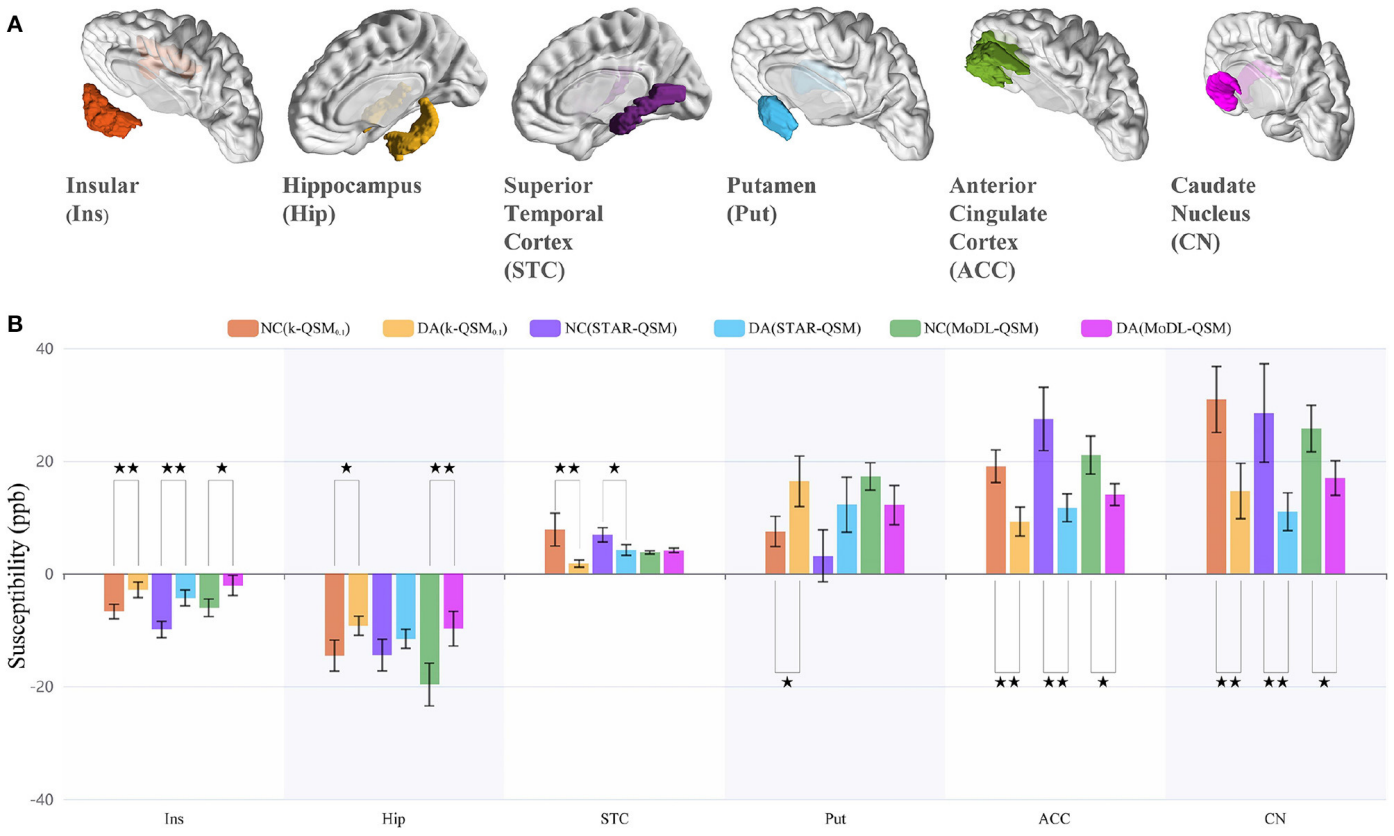
ROI analysis of the results of 15 healthy normal controls (NC) and 15 drug addicts (DA) using the k-QSM, STAR-QSM, and MoDL-QSM methods. **(A)** The selected quantitative susceptibility mapping (QSM) segmented region of interests (ROIs). **(B)** Susceptibility values of the ROIs, presented as mean ± standard deviation with ⋆⋆ denoting extremely significant (*p* < 0.01) and ⋆ sufficiently significant (0.01 ≤ *p* < 0.05) difference between mean values of the groups.

To further evaluate our proposed method for clinical applications, we compare different QSM reconstruction methods on in-house dataset to explore whether the QSM reconstructed by different methods can distinguish drug addicts (DA) and healthy normal controls (NC). Figure 9A displays six selected regions in 3D views, and Figure 9B illustrates the mean±standard deviation susceptibility values of each region calculated using k-QSM_0.1_, STAR-QSM, and MoDL-QSM methods, and *p*-values of the *t*-test for the means of two independent samples.

The results of k-QSM_0.1_ showed significant differences between the NC and DA in all six ROIs with 56.93% deficit (*p* = 0.0075) in the insular, 36.25% decline (*p* = 0.029) in the hippocampus, 77.60% reduction (*p* = 0.0075) in the STC, 51.62% shrinkage (*p* = 0.0014) in the ACC, 52.62% decrease (*p* = 0.0059) in the CN, and 119.54% increase (*p* = 0.025) in the putamen. Meanwhile, the results of STAR-QSM showed significant differences in insular (*p* = 0.00039), STC (*p* = 0.020), ACC (*p* = 0.00070), and CN (*p* = 0.0095), while the results of MoDL-QSM showed significant differences in insular (*p* = 0.021), hippocampus (*p* = 0.0058), ACC (*p* = 0.012), and CN (*p* = 0.018). Note that the magnetic susceptibility level should be compared with the absolute value.

There are differences among the results of different methods on the same data in one brain region. For the NC group, the results of k-QSM_0.1_ were significantly different from those of STAR-QSM only in the ACC (*p* = 0.017). For the DA group, only in the hippocampus dose k-QSM_0.1_ showed no significant difference (*p* = 0.66) with STAR-QSM, which means that the above methods show different performances on the data of drug addicts. As previously mentioned, the results of MoDL-QSM are too smooth to reveal richer details, with the standard deviation 10.52% less than the results of k-QSM_0.1_ and STAR-QSM on average.

According to previous studies (Franklin et al., 2002; Matochik et al., 2003; Thompson et al., 2004; Avants et al., 2007; Groman et al., 2013), the proposed k-QSM can detect the expected magnetic susceptibility changes between healthy subjects and drug addicts in specific brain regions. This is discussed in the next section.

## 4. Discussion

In this study, a deep-learning method, k-QSM, was developed to perform ill-conditioned dipole inversion of QSM reconstruction. The proposed k-QSM measures high-quality COSMOS-like QSM (Figure 3 and Table 1) and shows the ability to preserve the anisotropy of tissue susceptibility in WM (Figure 5). It achieves better performance with truncated value of 0.1 than 0.2 regarding the ability of generalization and anisotropy preserving. The trained models of k-QSM and other deep-learning methods were tested on the datasets acquired from different centers, and the trained k-QSM shows the potential of generalization, achieving a balance between reconstructing richer high-frequency details and suppressing artifacts. The k-QSM can also detect the expected variation in susceptibility from healthy to drug addicts in specific regions of the brain.

The results of k-QSM (both 0.1 and 0.2) are the most COSMOS-like with the lowest residual error, benefitting from the following: (a) in the function of MSE loss, the k-QSM mainly learns to fix ill-posed signals of TKD and retains a majority of correct signals of TKD with the action of L1 loss in non-ill-posed regions; (b) considering that the field-susceptibility relationship is intimate in the Fourier domain, learning in the Fourier domain can directly prevent correct signals from being confused, because a value in the space domain is superposed from all signals in the Fourier domain. Thus, if learned in the space domain, all signals in the Fourier domain are involved and a correct signal can turn out to be improper. In addition, the methods (QSMnet^+^, LP-CNN, and MoDL-QSM) trained in the space domain have to calculate the model loss in the Fourier domain, which may result in error accumulation. Thus, the proposed k-QSM needs less to learn than previous deep-learning methods and maintains numerous correct signals in TKD, so that the k-QSM can reconstruct QSM with less residual errors.

The proposed k-QSM can preserve the MSA from MRI phase measurements in WM as well as TKD, which calculates QSM directly from phase measurements so that the MSA is preserved (Figure 5). As mentioned above, the k-QSM retains many correct signals from TKD, which comprise tissue anisotropy information. Therefore, k-QSM can also preserve the MSA, although the training process takes COSMOS as a label. As shown in Table 2, k-QSM_0.1_ preserves more MSA than k-QSM_0.2_, because k-QSM_0.1_ keeps more signals from TKD than k-QSM_0.2_. The previous COSMOS-labeled deep-learning methods, for example, QSMnet+, cannot retain the MSA information because COSMOS assumes isotropic magnetic susceptibility (Lee et al., 2010; Li W. et al., 2012; Wharton and Bowtell, 2012) and they learn to reconstruct COSMOS-like QSM completely. The tissue MSA is reflected in different sampling orientations. Meanwhile, MoDL-QSM takes susceptibility tensor terms (χ_13_, χ_23_ and χ_33_) as labels to follow the susceptibility tensor imaging (STI) physical model, and as a result the MSA was preserved (Feng et al., 2021). Dmitriy et al. inferred that the biological tissue structure anisotropy influenced the calculation of susceptibility and the MSA of tissue in WM, and the MSA provided a quantitative interpretation of data from MR phase imaging of WM diseases (Yablonskiy and Sukstanskii, 2017). Thus, the ability to maintain the MSA of k-QSM is important for the clinicopathologic diagnosis of WM diseases. Learn Less means that the k-QSM only learns to fix the ill-posed signals in TKD around zero values of the dipole kernel and Infer More indicates that the results of k-QSM are both COSMOS-like and anisotropic in tissue susceptibility.

Additionally, the proposed k-QSM demonstrates strong generalization ability on datasets not used in training, including healthy and patient datasets from different acquisition sites. The healthy dataset was measured by 3T scanner (Siemens) and the patient dataset included data of patients with multiple sclerosis and hemorrhage acquired with a 3T GE HDxt MR scanner. Even though the k-QSM model is trained on the 7T dataset, it still works well on these 3T datasets. The utilization of the dropout layer in the deep neural network makes it possible for k-QSM to prevent overfitting (Srivastava et al., 2014) and enhance the ability of generalization (Dahl et al., 2013). Besides, as mentioned above, k-QSM keeps the correct signals in the Fourier domain from TKD. In the meanwhile, k-QSM inherits generalization ability from TKD, which works well for all datasets as an analytical solution with numerous correct signals, and, thus, k-QSM_0.1_ shows stronger generalization ability than k-QSM_0.2_. What is more, the k-QSM method is trained in k-space directly, and expresses the field–susceptibility relationship as Equation (2) described, thus, the trained k-QSM model can be applied to almost arbitrary dataset to process QSM dipole inversion.

Furthermore, the proposed k-QSM can also reconstruct QSM on dataset of drug addiction and reveal the impairment of drug administration through susceptibility changes in brain tissues between DA and healthy NC groups. Consistent with the reported decreases in gray matter concentration in the anterior cingulate, superior temporal cortices and anteroventral insular of cocaine abusers (Franklin et al., 2002; Matochik et al., 2003), smaller hippocampal volumes of MA abusers (Thompson et al., 2004), and smaller caudate volumes of cocaine abusers (Avants et al., 2007), the k-QSM detected the debilitation of susceptibility in the insular, hippocampus, STC, ACC, and CN, as shown in Figure 9. One potential interpretation is that a reduction in gray matter concentration or volume can decrease the level of magnetic susceptibility. Besides, the k-QSM can observe the increase of susceptibility in putamen in accord with the research of methamphetamine-induced increases in putamen gray matter (Groman et al., 2013). Thus, k-QSM is more sensitive to the susceptibility change caused by drug addiction, which can provide a potential biomarker for evaluating different treatment strategies for drug addicts.

Although the proposed k-QSM can measure COSMOS-like susceptibility distribution with rich high-frequency details and immensely suppress artifacts compared to TKD, there are still a few artifacts left. One potential interpretation is that because trained in the Fourier domain, the k-QSM cannot remove the artifacts explicitly in the spatial domain. In addition, the training of COSMOS labels still suffers from artifacts because sub-optimal orientations cannot provide sufficient conditions to stabilize the inversion problem. Further studies could design more elaborate loss functions, such as gradient loss in the spatial domain, to improve the k-QSM, but that would be concerning because the utilization of gradient loss function might smoothen the results and lead to loss of anisotropy of tissue susceptibility. Besides, we only conducted two truncated values to verify the learning performance of k-QSM, more thresholds should be tested to seek for the optimal truncated value in k-QSM method.

## Data Availability Statement

The raw data supporting the conclusions of this article will be made available by the authors, without undue reservation.

## Ethics Statement

Ethical review and approval was not required for the study on human participants in accordance with the local legislation and institutional requirements. The patients/participants provided their written informed consent to participate in this study.

## Author Contributions

JH and YC: study conception and design. YC, LW, RW, and JH: acquisition of data. YC, LW, RW, and YZ: analysis and interpretation of data. JH: drafting of the manuscript. JH, LW, RW, and YZ: critical revision. All authors contributed to the article and approved the submitted version.

## Funding

This work was partially funded by the National Nature Science Foundations of China (Grant No.62161004), the Guizhou Provincial Scientific Research Project ZK[2021]key 002, the Program PHC-Cai Yuanpei 2018 (No.41400TC), and the International Research Project METISLAB.

## Conflict of Interest

The authors declare that the research was conducted in the absence of any commercial or financial relationships that could be construed as a potential conflict of interest.

## Publisher's Note

All claims expressed in this article are solely those of the authors and do not necessarily represent those of their affiliated organizations, or those of the publisher, the editors and the reviewers. Any product that may be evaluated in this article, or claim that may be made by its manufacturer, is not guaranteed or endorsed by the publisher.
